# Activation of the Medial Prefrontal Cortex Reverses Cognitive and Respiratory Symptoms in a Mouse Model of Rett Syndrome

**DOI:** 10.1523/ENEURO.0277-17.2017

**Published:** 2018-01-10

**Authors:** C. James Howell, Michael P. Sceniak, Min Lang, Wenceslas Krakowiecki, Fatimah E. Abouelsoud, Saloni U. Lad, Heping Yu, David M. Katz

**Affiliations:** Department of Neurosciences, Case Western Reserve University School of Medicine, Cleveland, OH 44106

**Keywords:** autism spectrum disorder, DREADD, hypofrontality, Mecp2, memory, mPFC

## Abstract

Rett syndrome (RTT) is a severe neurodevelopmental disorder caused by loss-of-function mutations in the gene encoding methyl-CpG-binding protein 2 (MeCP2; Amir et al., 1999), a transcriptional regulatory protein (Klose et al., 2005). Mouse models of RTT (*Mecp2* mutants) exhibit excitatory hypoconnectivity in the medial prefrontal cortex (mPFC; Sceniak et al., 2015), a region critical for functions that are abnormal in RTT patients, ranging from learning and memory to regulation of visceral homeostasis (Riga et al., 2014). The present study was designed to test the hypothesis that increasing the activity of mPFC pyramidal neurons in heterozygous female *Mecp2* mutants (Hets) would ameliorate RTT-like symptoms, including deficits in respiratory control and long-term retrieval of auditory conditioned fear. Selective activation of mPFC pyramidal neurons in adult animals was achieved by bilateral infection with an AAV8 vector expressing excitatory hm3D(Gq) DREADD (Designer Receptors Exclusively Activated by Designer Drugs) (Armbruster et al., 2007) under the control of the CamKIIa promoter. DREADD activation in *Mecp2* Hets completely restored long-term retrieval of auditory conditioned fear, eliminated respiratory apneas, and reduced respiratory frequency variability to wild-type (Wt) levels. Reversal of respiratory symptoms following mPFC activation was associated with normalization of Fos protein levels, a marker of neuronal activity, in a subset of brainstem respiratory neurons. Thus, despite reduced levels of MeCP2 and severe neurological deficits, mPFC circuits in Het mice are sufficiently intact to generate normal behavioral output when pyramidal cell activity is increased. These findings highlight the contribution of mPFC hypofunction to the pathophysiology of RTT and raise the possibility that selective activation of cortical regions such as the mPFC could provide therapeutic benefit to RTT patients.

## Significance Statement

Rett syndrome (RTT) is a devastating disorder for which no treatments are currently available beyond supportive care. Although significant progress has been made in understanding RTT pathophysiology at the molecular and cellular levels, the relationship between dysfunction in specific brain circuits and the expression of specific RTT symptoms remains unclear. The present study provides the first direct evidence of a link between hypoactivity in the mPFC and cognitive and respiratory symptoms in *Mecp2* mutants by demonstrating that activation of the mPFC restores wild-type (Wt) function in these domains. Thus, in addition to highlighting the contribution of mPFC dysfunction to the pathophysiology of RTT, these findings raise the possibility that targeted activation of specific cortical regions could provide therapeutic benefit to RTT patients.

## Introduction

Rett syndrome (RTT) is caused by loss-of-function mutations in the gene encoding methyl-CpG-binding protein 2 (MeCP2) and is one of the most physically debilitating disorders on the autism spectrum. RTT patients exhibit a complex constellation of symptoms ranging from deficits in motor function and cognition to dysregulation of breathing and autonomic control (Amir et al., 1999). Studies in RTT mouse models, which recapitulate the symptomatology of human RTT, as well as human postmortem studies have revealed that loss of *Mecp2* does not result in neuronal degeneration or cell loss (Akbarian, 2003) but rather in abnormalities in the structure and function of brain microcircuits (Shepherd and Katz, 2011). These changes include marked alterations in synaptic strength and connectivity (Katz et al., 2016) which differ among brain regions and appear to be reversible (Guy et al., 2007; Robinson et al., 2012). One of the most striking effects of *Mecp2* loss on brain circuit function is a decrease in excitatory synaptic connectivity in the motor, somatosensory, visual, and midline limbic cortices, including the medial prefrontal cortex (mPFC; Katz et al., 2016). Cortical hypoconnectivity is associated with multiple factors, including reduced density and maturity of dendritic spines on pyramidal neurons (Chao et al., 2007; Belichenko et al., 2009; Kishi and Macklis, 2010; Stuss et al., 2012; Sceniak et al., 2015), a shift in the balance of excitatory and inhibitory synaptic signaling molecules toward decreased excitation (Durand et al., 2012; Sceniak et al., 2015) and, in some cases, increased inhibitory connectivity (Durand et al., 2012). As a result, many cortical regions in the *Mecp2* mutant brain are hypoactive at rest compared to wild-type (Wt) controls (Kron et al., 2012).

Hypoactivity of pyramidal neurons in the mPFC in *Mecp2* mutants is of particular interest given the role of the mPFC in multiple brain functions that are abnormal in RTT, ranging from learning and memory to respiratory and autonomic homeostasis. Despite this, the role of mPFC dysfunction in the pathophysiology of RTT has been little explored. For example, the ventral mPFC, or “visceral cortex” (Neafsey, 1990; Hassan et al., 2013), is responsible for regulating behavioral state-dependent changes in respiratory and autonomic homeostasis, as during stress or in response to conditioned learning (Frysztak and Neafsey, 1991; Alexandrov et al., 2007). Structures in the ventral mPFC, including the prelimbic (PL), infralimbic (IL), and dorsal peduncular cortex (dPC) give rise to extensive direct projections to cardiorespiratory cell groups in the pons and medulla, as well as indirect projections to subcortical forebrain cell groups that project to the brainstem, including the hypothalamus and amygdala (Gabbott et al., 2005). On the basis of these observations, we hypothesize that hypofunction of the mPFC may influence cardiorespiratory control in RTT. This possibility is supported by the fact that abnormal breathing in RTT worsens with stress and can be nearly normal during sleep, arguing strongly for a significant behavioral component (Weese-Mayer et al., 2008; Ren et al., 2012).The ventral mPFC has also been shown to be required for cognitive tasks, such as fear memory consolidation and retrieval (Sierra-Mercado et al., 2006, 2011), that are disrupted in RTT. For example, *Mecp2* mutants are impaired in their ability to retain memory of auditory conditioned fear 24 h after training (Tai et al., 2016), a task that requires ongoing activity in the PL (Corcoran and Quirk, 2007) and its projections to the dorsal midline thalamus (dMT; Adhikari et al., 2015). Therefore, the present study was designed to determine whether or not increasing activity in mPFC pyramidal neurons would ameliorate symptoms of cognitive impairment as well as abnormal breathing in *Mecp2* mutant mice.

## Materials and Methods

### Animals

All experiments were performed on female *Mecp2^tm1.1Jae^* Wt and Het mice on a 129S/BalbC/Bl6 background, and each animal was genotyped before and after every experiment to confirm genetic identity.

### Surgery and viral constructs

Animals (8-10 weeks of age) were sedated with isoflurane (5% for induction, 1.5-0.8% for maintenance), fixed in a stereotaxic frame (Stoelting) and then subjected to bilateral craniotomy at bregma +1.75 mm, ±0.25 mm from the midline. Infusions of 500-nl viral construct (AAV8-CamKIIa-hm3D-mCherry; UNC vector core) were made at a depth of 2.0 mm using a Hamilton NeuroS syringe. For injections in the motor cortex, infusions were targeted at bregma +1.9 mm, 1.5 mm from the midline, and 0.1 mm below the dorsal surface of the brain. To reduce backflow of the injectate along the needle path, infusions were performed at a rate of 100 nl per 2 min with an additional 2 min following the final 100 nl. After surgery, animals were returned to their cages for two weeks to allow for viral expression.

### Designer Receptors Exclusively Activated by Designer Drugs (DREADD)-Gq Expression Mapping

**Figure 1.**
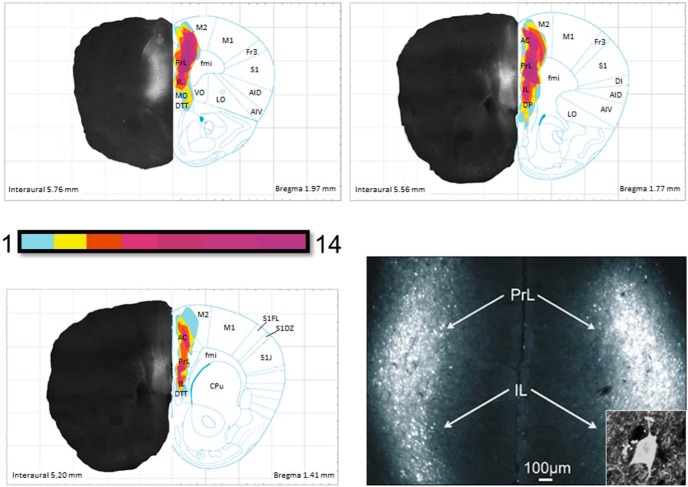
DREADD-Gq expression in the mPFC. The distribution of DREADD-Gq-labeled neurons was plotted from low-magnification micrographs of mCherry labeling in serial coronal sections through the forebrain in 14 animals; the plots were then overlaid to produce heat maps of the injection sites in which the color coding represents the extent of overlap in the distribution of labeled neurons in all 14 animals. These heat maps are shown on the right side of each figure, with representative hemi-brain sections shown on the opposite side (note that all animals received bilateral injections). The faint labeling seen lateral to the mPFC at 1.77 and 1.41 mm rostral to bregma is mCherry expression in the axons of infected neurons. Bottom right, Representative photomicrograph of mCherry-labeled neurons in a coronal section through the mPFC of an infected animal. Anatomic labels for this and all subsequent figures as follows: AC, anterior cingulate; AID, dorsal agranular insular cortex; AIV, ventral agranular insular cortex; CPu, caudate putamen; DI, dysgranular insular cortex; DP, dorsal peduncular cortex; DTT, dorsal tenia tecta; fmi, forceps minor of the corpus callosum; Fr3, frontal cortex area 3; IL, infralimbic cortex; LO, lateral orbital cortex; M1, primary motor cortex; M2, secondary motor cortex; MO, medial orbital cortex; PrL, prelimbic cortex; S1, primary somatosensory cortex; S1DZ, somatosensory cortex dysgranular zone; S1FL, forelimb region of the somatosensory cortex; S1J, jaw region of the somatosensory cortex; VO, ventral orbital cortex. Inset shows a high-magnification view of an infected neuron.

To verify the distribution of DREADD-Gq labeling following injection (Fig. 1), low-magnification images of mCherry expression from a representative cohort of 14 injected animals (seven Wt and seven Het) were taken using a Zeiss Axiophot. These images were montaged using Adobe Photoshop, then overlaid onto coronal atlas images (Franklin and Paxinos, 2012). We then used Corel Draw tracing and lenses to create heat maps of DREADD-Gq expression showing the extent of overlap among all 14 animals. Additionally, these images were used to quantify the number of infected cells in the mPFC in *Mecp2* Wt and Het mice. ImageJ was used to select and quantify the number of cells in a 200-µm^2^ circular area surrounding each injection site. Data were collected from three tissue sections spanning the rostrocaudal extent of the injection sites from each of five animals per genotype.

### Electrophysiology

*In vivo* extracellular recordings were made in a subset of mPFC-DREADD-Gq animals, two weeks after infection. Animals were anesthetized with urethane (1000 mg/kg) and placed in a stereotaxic apparatus (Stoelting). Craniotomies were made as described above and tungsten sharp electrodes (AM-Systems) were lowered into the mPFC at the same coordinates used for virus injections. Filtered extracellular recordings (1 kHz high-pass) were amplified (AM-Systems) and digitally stored on a computer running custom software (Matlab) through an A/D converter (National Instruments). Extracellular recordings of spontaneous activity within the mPFC were collected under urethane anesthesia while simultaneously monitoring respiration. Recordings of spontaneous activity were collected during the baseline condition for 10 min, and then 45 min after administration of clozapine-N-oxide (CNO; 0.03 mg/kg, i.p.) to determine the effects of mPFC-DREADD-Gq activation on spiking activity. All animals were perfused after recordings to perform a histologic reconstruction of the recording site and to confirm the expression of the mCherry tag associated with the AAV8-hm3-DREADD construct.

### Plethysmography

Breathing analysis was performed on unrestrained mice using whole-body plethysmographs (EMMS) in which constant air flow was provided at 1 l/min. Analysis was restricted to periods of quite breathing, defined as times when the animal was not involved in ambulation, grooming, or investigatory sniffing and all four paws were resting on the chamber floor. Animals were treated with either saline or CNO (0.03 mg/kg, i.p.) and then allowed to acclimate to the chamber for 1 h. After acclimation, respiratory data were gathered over the next 2–3 h. eDaq software (EMMS) was used to analyze the respiratory data for frequency, coefficient of frequency variation (CV), and apneas/min (where an apnea was defined as a respiratory pause lasting at least twice the duration of the average expiratory time, Te). Each animal served as its own control, being first injected with saline before plethysmography, then with CNO 24–48 h later, again followed by plethysmography. In addition, in one cohort of animals, respiration was measured a third time, 24–48 h after CNO injection and 1 h following saline treatment.

### Cue-dependent fear conditioning

Animals were habituated in a fear conditioning chamber (Med Associates) for 90 s before being exposed to four sequential pairings of a 5 kHz, 80 dB tone for 30 s (conditioned stimulus; CS) which coterminated with a 0.5-mA foot shock (unconditioned stimulus; US); each pairing was separated by 30-s interstimulus intervals and the time spent immobile during the CS presentation is reported as % freezing. Animals that did not learn the CS-US pairing, i.e., that did not exhibit increased freezing during conditioning, were excluded from further analysis. To assess short-term memory (STM) retrieval (4 h after CS-US pairing), animals were exposed to the CS alone, repeated twice with a 30-s interval, and the % freezing during the two CS exposures was averaged. These data are displayed as a percentage of the freezing to CS4 during conditioning ([Freezing_Tone(LTM or STM)_ * 100]/Freezing_CS4_). Long-term memory retrieval was tested 24 h after fear conditioning using the same protocol as described for STM retrieval testing. To avoid environmental context as a confounding variable, both STM and long-term memory (LTM) testing were performed in a novel environment produced by modifying the fear conditioning chamber with a white floor, black a-frame insert, black and white striped background, and a vanilla scent. Some of the animals used for fear conditioning were heterozygous carriers of *Thy1-EGFP^MJrs/J^* that were generated by crossing homozygous male *Thy1-EGFP^MJrs/J^* mice on a Bl6 background with heterozygous female *Mecp2^tm1.1Jae^*mice from our main colony. The presence of the transgene was incidental to the present study; these animals were included to increase group sizes during a period of limited animal availability and did not differ in performance from the main colony stock on either fear memory acquisition or short- and long-term fear memory retrieval (p = 0. 49, *p* = 0.51, *p* = 0.92, respectively by Student’s *t* test, comparing animals with and without the transgene).

### Immunohistochemistry

After behavioral testing, mice were deeply anesthetized with isoflurane and then perfused with 0.9% saline, followed by 4% paraformaldehyde (PFA) by cardiac perfusion. PFA fixed brains were sectioned at 40 µm on a cryostat microtome (Jung Frigocut 2800N). Free floating sections were exposed to a primary antibody directed against either mCherry (Life technologies M11217; 1:500) or Fos protein (SySy 226003; 1:3000) overnight at room temperature. The next day, the tissue was incubated sequentially with a biotinylated secondary antibody (Jackson #112-065-003; 1:400 or Vector BA1000), ABC kit (Vector), and developed with the Sigmafast diaminobenzidine kit. Mounted sections were imaged using a light microscope (Zeiss Axiophot) and photographed with a CCD camera (Q-Imaging).

### Blinding and randomization

Animals were selected for all experiments, and assigned to experimental groups without prior knowledge of phenotypic severity. For fear conditioning experiments and Fos-labeling studies, the investigators were blind to genotype and treatment group at all stages including data analysis. Blinding for treatment group was not necessary for plethysmography and electrophysiology experiments because each animal received both saline and CNO and thereby served as its own control.

### Statistics

Minimum group sizes were determined using G*Power 3 power analysis software (Faul et al., 2007). Each data set was tested for normality using the Shapiro–Wilk test in the Statistical Package for Social Sciences (SPSS), and parametric tests were performed either on the raw data for normally distributed data sets or on log transformed data for non-normally distributed data sets.

## Results

### DREADD expression in mPFC pyramidal neurons

To enable selective activation of mPFC pyramidal neurons, we injected an AAV8 viral construct expressing the excitatory DREADD AAV8-CamKIIa-hm3D-mCherry (DREADD-Gq; UNC Vector Core) bilaterally into the mPFC of female Wt and *Mecp2*^tm1.1Jae/+^ (Het) littermates (bregma +1.75 mm, ±0.25 mm from the midline, −2.00 mm depth). Following injection of the viral construct, two weeks were allowed for the expression of DREADD-Gq in the infected neurons before beginning subsequent experimental protocols. Reconstruction of the injection sites from 14 animals revealed that our injection coordinates generated bilateral infection of neurons in the pre- and infra-limbic cortices (PL and IL, respectively) with some spread into the dPC cortex ventrally, and the cingulate (CG) and motor cortices (MCtxs) dorsally (Fig. 1; see Methods for details). Animals that displayed either unilateral expression or expression surrounding the lateral ventricles were eliminated from further analysis. To compare the efficiency of viral transduction in Wt and Het animals, we estimated the number of DREADD-mCherry positive cells in the injection site in a subset of animals (see Materials and Methods for details) and found no difference between genotypes (Wt, 106.13 ± 8.59 vs null, 106.233 ± 11.13). Consistent with previous evidence that AAV8 is not transported either transynaptically or in the retrograde direction (Aschauer et al., 2013), we found no cell bodies labeled with DREADD-Gq outside of the regions mentioned above.

**Figure 2.**
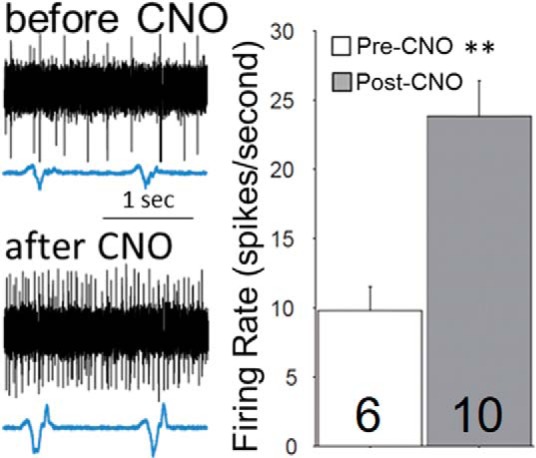
Activation of mPFC pyramidal neurons by DREADD-Gq increases neuronal activity *in vivo*. Left, Representative extracellular traces of spontaneous activity in the mPFC of mPFC-DREADD-Gq Het animals before and after CNO treatment. Right, Summary data from two animals; the total number of recorded cells is shown within each bar. **p < 0.01 by Wilcoxon rank sum test.

To verify that CNO injection altered neuronal activity in the mPFC of mPFC-DREADD-Gq-infected Het mice, we analyzed *in vivo* extracellular recordings of spiking activity before and after CNO administration (0.03 mg/kg, i.p.) in anesthetized animals. These experiments revealed that the firing rate of neurons in the mPFC increased >2-fold after CNO (average firing rate 19.67 ± 3.43 pre-CNO vs 47.60 ± 5.20 post-CNO; Wilcoxon rank sum test; Fig. 2), consistent with previous studies (Armbruster et al., 2007; Alexander et al., 2009; Ferguson et al., 2011; Krashes et al., 2011; Atasoy et al., 2012; Kong et al., 2012; Stachniak et al., 2014; Chen et al., 2015; Scofield et al., 2015; Urban and Roth 2015; Vardy et al., 2015).

### DREADD-positive fibers project to brain regions important for respiratory control and cue-dependent fear memory

**Figure 3.**
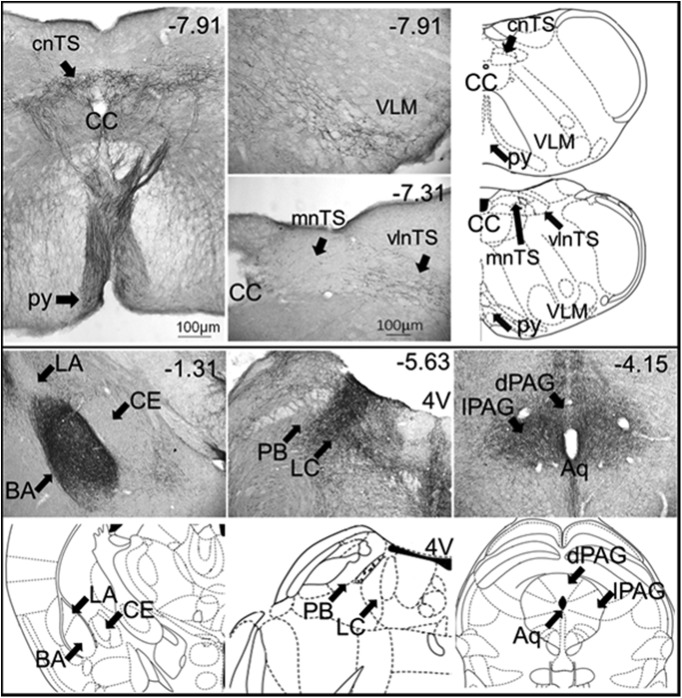
Photomicrographs showing mCherry-positive fibers in coronal sections through brain regions important for respiratory control and cue-dependent fear memory following DREADD-Gq infection of the mPFC. Numbers in the top right of each micrograph correspond to the distance from bregma (mm). 4V, fourth ventricle; Aq, aqueduct of Sylvius; BA, basal amygdala; CC, central canal; CE, central amygdala; cnTS, commissural nTS; LC, locus coeruleus; LA, lateral amygdala; mnTS, medial nTS; PB, parabrachial nucleus; py, pyramidal tract; vlnTS, ventrolateral nTS (abbreviations from Franklin and Paxinos, 2012).

To begin elucidating how activation of the mPFC might impact *Mecp2* mutant respiratory and cognitive phenotypes, we asked whether or not mPFC-DREADD-Gq-infected neurons project to regions known to be important for either respiratory control or cue-dependent fear memory. To approach this issue, we took advantage of the fact that the mCherry tag in the DREADD-Gq construct can be visualized throughout the infected neurons, including their axonal projections, and amplified by immunocytochemical staining. Analysis of brain sections from mPFC-DREADD-Gq-infected animals revealed extensive projections of mCherry-labeled axons within numerous components of the ponto-medullary respiratory network, including the parabrachial nucleus and locus coeruleus in the pons, and the caudal raphe, nucleus of the solitary tract (nTS) and ventrolateral reticular formation in the medulla (Fig. 3). Corticobulbar projections to the medulla could be clearly seen exiting the pyramidal tract before ramifying dorsally in the caudal raphe and nTS and laterally in the ventrolateral medulla (VLM; Fig. 3). Fibers were also present in mid- and forebrain structures that influence breathing and project to the ponto-medullary network, including the periaqueductal gray (PAG), hypothalamus, and the basolateral nucleus of the amygdala (BLA), a structure which is also critically important for cue-dependent fear memory consolidation.

### Increasing mPFC pyramidal neuron activity restores normal respiration in *Mecp2* mutants

**Figure 4.**
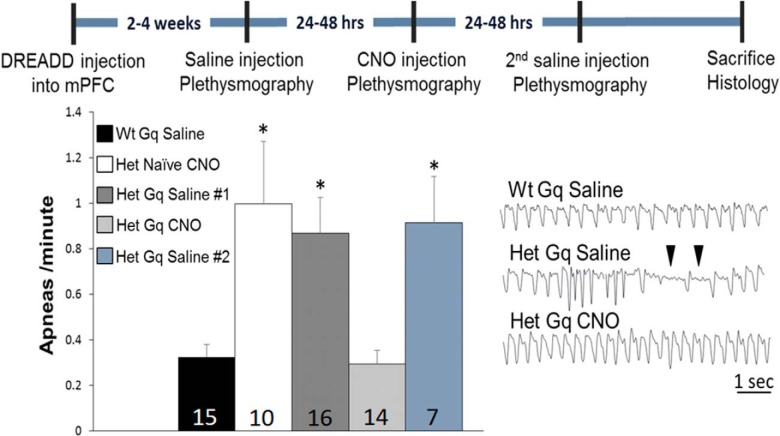
DREADD-Gq activation of pyramidal neurons in the mPFC eliminates the apneic breathing phenotype in *Mecp2* mutants. The timeline of DREADD injection, CNO (or saline) treatment and plethysmographic recordings is shown at the top of the figure. The bar graph shows summary data illustrating the apneas/min for each experimental group with group sizes included within each bar. *p < 0.05 by ANOVA with Bonferroni *post hoc* test. Bottom right, Representative respiratory traces; ▼ indicates typical respiratory pauses scored as apneas.

To determine whether or not changes in the activity of mPFC pyramidal neurons would alter *Mecp2* mutant respiratory phenotypes, we compared breathing in mPFC-DREADD-Gq-infected Wt and Het animals 1 h after injection of either saline (control) or the DREADD ligand CNO, using whole-body plethysmography (Fig. 4, experimental timeline). In a subset of animals, saline was also given 24-48 h after CNO, and animals were again tested by plethysmography to determine the reversibility of any observed effects of the DREADD ligand. To address the possibility that CNO may have nonspecific effects on respiration (Loffler et al., 2012; Gomez et al., 2017), we also analyzed the respiratory response to CNO in Hets that had not been infected with DREADD-Gq (naïve Hets).

**Figure 5.**
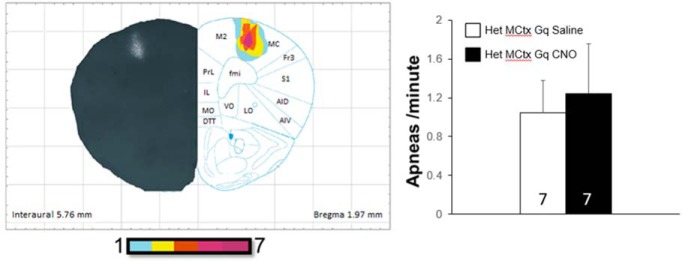
Activation of pyramidal neurons in the motor cortex by DREADD-Gq does not alter respiration. Left, The distribution of DREADD-Gq-labeled neurons was plotted from low-magnification micrographs of mCherry labeling in coronal sections through the forebrain in all animals tested; the plots were then overlaid to produce a heat map of the injection sites in which the color coding represents the extent of overlap in the distribution of labeled neurons in all seven animals. Note that all animals received bilateral injections. Right, Animals expressing DREADD-Gq in the motor cortex were subjected to two consecutive days of plethysmographic recording of respiration, 1 h following either saline treatment (day 1) or CNO treatment (day 2). The bar graph shows summary data illustrating the apneas/min on day 1 (saline) and day 2 (CNO), with group sizes included within each bar.

As previously described for *Mecp2* mutants (Katz et al., 2016), naïve Hets treated with CNO, as well as mPFC-DREADD-Gq-infected Hets treated with saline exhibited significantly more apneas than Wt animals (ANOVA with Bonferroni *post hoc* test; Fig. 4). However, in mPFC-DREADD-Gq-infected Hets treated with CNO, the apnea score was reduced to Wt levels (apneas/min; Wt Gq saline, 0.33 ± 0.16; Het Gq saline, 0.87 ± 0.15; Het Gq CNO, 0.29 ± 0.06; Fig. 4). This effect was completely reversible as mPFC-DREADD-Gq-infected Het mice injected with saline 24-48 h after receiving CNO exhibited the same level of apnea as they did before CNO (Het Gq saline #2, 0.91 ± 0.20; Fig. 4). To determine whether or not CNO activation of neurons infected by spread of the virus into the MCtx contributed to the apnea rescue in mPFC-DREADD-Gq-infected Hets, a parallel series of experiments was performed in animals in which DREADD-Gq infection was restricted to the MCtx. CNO injection had no effect on the number of apneas in these animals (Student’s *t* test; Het MCtx saline, 1.04 ± 0.34; Het MCtx CNO, 1.24 ± 0.52; Fig. 5). Together, these data demonstrate that activation of excitatory neurons in the mPFC can eliminate the apnea phenotype in *Mecp2* Hets.

**Figure 6.**
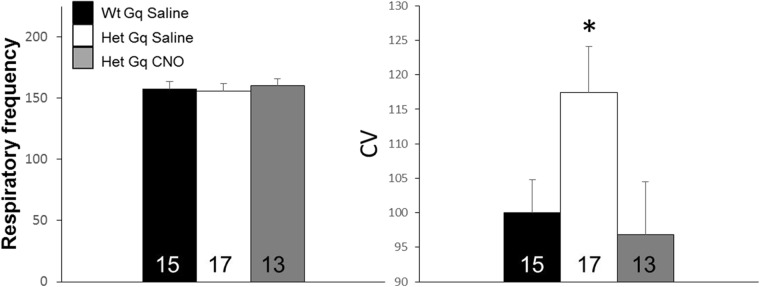
DREADD-Gq activation of pyramidal neurons in the mPFC reduces respiratory frequency variability to Wt levels without impacting the average frequency of respiration. Left, Summary data showing the mean instantaneous respiratory frequency for each experimental group. Right, Summary data showing the coefficient of variation (CV) for instantaneous respiratory frequency for each experimental group. Group sizes are shown within each bar. *p < 0.05 by ANOVA with LSD *post hoc* test.

We next sought to determine whether CNO treatment of animals expressing DREADD-Gq in the mPFC impacted other respiratory phenotypes previously observed in *Mecp2* mutant mice (Katz et al., 2016). Therefore, we analyzed both instantaneous respiratory frequency and the CV. Although we found no effect of either genotype or treatment on respiratory frequency (Fig. 6), CV was significantly increased in DREADD-Gq Hets treated with saline compared to DREADD-Gq Wt controls (Wt Gq saline, 100.00 ± 4.77, Het Gq saline, 117.41 ± 6.71 CV; Fig. 6). In contrast, CNO treatment of Hets expressing DREADD-Gq in the mPFC restored CV to Wt levels (Het Gq CNO, 96.85 ± 7.60 CV; ANOVA with LSD *post hoc* test; Fig. 6). The increase in CV in Het mice, and the subsequent CV rescue following CNO treatment could be attributed to the effects of genotype and treatment, respectively, on the number apneic pauses, as these effects disappeared when CV was calculated with apneas removed (data not shown).

### DREADD activation of mPFC pyramidal neurons impacts downstream function in respiratory-related neurons

**Figure 7.**
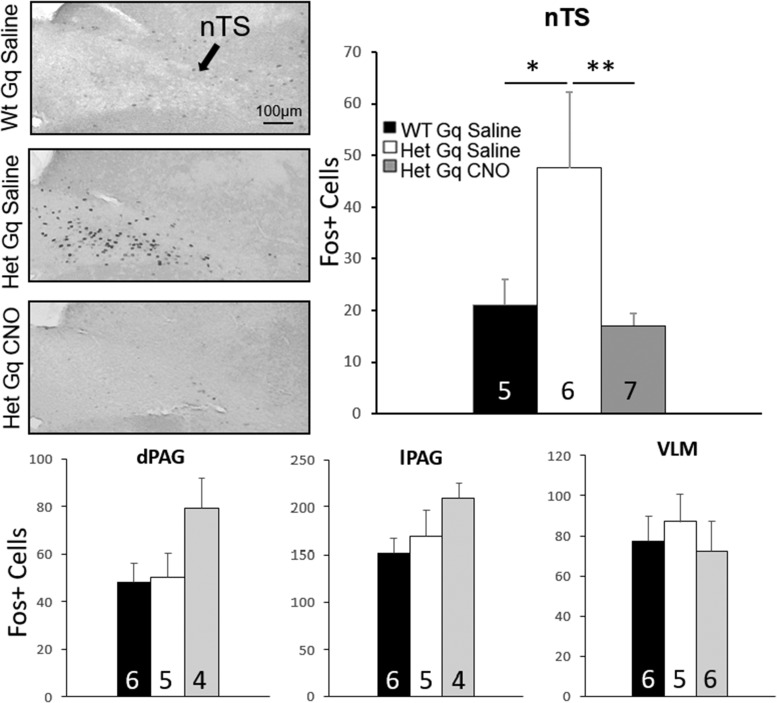
Activation of mPFC pyramidal neurons by DREADD-Gq impacts neuronal function in respiratory subnuclei of the nTS. Animals were sacrificed 90 min after either saline CNO injection and processed for Fos immunostaining. Photomicrographs show representative sections through the caudal nTS from 3 different animals, each of which received bilateral injections of DREADD-Gq in the mPFC. CNO treatment restored Fos levels in the nTS of mPFC-DREADD-Gq Het mice to Wt levels. *p < 0.05, **p < 0.01, ANOVA with LSD *post hoc* test. No significant differences were observed between treatment groups in the dPAG, lPAG or VLM.

To determine whether activation of the mPFC in *Mecp2* mutants altered neuronal activity in downstream respiratory targets, we used immunocytochemical staining to compare expression of Fos protein in response to CNO treatment in mPFC-DREADD-Gq-infected Wt and Het animals. We previously showed that Fos, the protein product of the activity-dependent immediate early gene, cFos, is a sensitive and accurate marker of differences in neural circuit activity between Wt and *Mecp2* mutant mice (Kron et al., 2012). We focused in particular on the respiratory subnuclei of the nTS, which exhibit synaptic hyperexcitability and markedly increased Fos expression in *Mecp2* mutants compared to Wt (Kron et al., 2012). Synaptic hyperexcitability in respiratory subnuclei of nTS, including those involved in regulation of the inspiratory off-switch through the Hering-Breuer reflex pathway, is thought to be a substrate for respiratory hyperreflexia in *Mecp2* mutants and may thereby contribute to apneic breathing (Kron et al., 2012; Dhingra et al., 2013). In the present study, CNO treatment of mPFC-DREADD-Gq-infected Het mice reduced Fos expression in nTS subnuclei to Wt levels (average Fos+ expression Wt Gq saline, 19.56 ± 5.76, Het Gq saline, 45.15 ± 15.06, Het Gq CNO, 14.59 ± 2.68; ANOVA with LSD *post hoc* test; Fig. 7), indicating that activation of mPFC pyramidal neurons can impact neuronal function in downstream respiratory targets. Similar changes were not seen in other brainstem respiratory cell groups, including the VLM and PAG [dorsal PAG (dPAG); Wt Gq saline, 48.23 ± 8.11, Het Gq saline, 50.38 ± 9.83; Het Gq CNO, 79.32 ± 12.58; lateral PAG (lPAG); Wt Gq saline, 152.58 ± 14.19, Het Gq saline, 169.51 ± 27.71; Het Gq CNO, 209.24 ± 16.34; VLM; Wt Gq saline, 70.19 ± 12.32, Het Gq saline, 86.99 ± 13.60; Het Gq CNO, 72.43 ± 14.67; Fig. 7], although there was a trend toward increased expression in the dPAG in the Het Gq CNO group.

### DREADD activation of mPFC pyramidal neurons restores long-term retrieval of auditory conditioned fear

**Figure 8.**
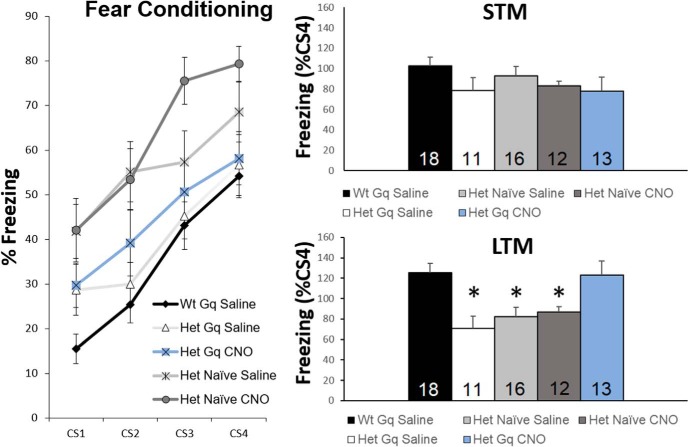
Activation of mPFC pyramidal neurons by DREADD-Gq rescues long-term expression of cue-dependent fear memory. Left, Conditioning to CS-US pairings across four trials demonstrates similar responses in all groups using % freezing (immobility) during the CS presentations as an index of fear learning. Right, Short-term (4 h after conditioning) and long-term (24 h after conditioning) memory retrieval were tested by exposure to the CS alone, with the animal in a test chamber that was distinguished from the conditioning chamber by novel environmental features (see Materials and Methods). Data are presented as a percentage of the freezing to CS4 during conditioning ([Freezing_Tone(LTM or STM)_ * 100]/Freezing_CS4_). *p < 0.05 by ANOVA with LSD *post hoc* test.

In addition to cortical modulation of visceral homeostasis, the mPFC plays critical roles in cognition that are mediated through projections to diverse cortical and subcortical cell groups (Vertes, 2004; Gabbott et al., 2005). In particular, direct projections from mPFC pyramidal neurons to the BLA mediate the consolidation of auditory conditioned fear memory following conditioned fear learning, and the short-term retrieval of this memory while mPFC projections to the dMT mediate long-term retrieval (Do-Monte et al., 2015). The following series of experiments was designed to determine whether or not hypoactivity in the mPFC was associated with a deficit in retrieval of auditory conditioned fear memory and, if so, whether the deficit is reversible by DREADD activation of mPFC pyramidal neurons. To approach these issues, we compared acquisition and retrieval of auditory conditioned fear memory among five groups of animals; noninfected Het control animals given either saline or CNO (0.03 mg/kg, i.p.), mPFC-DREADD-Gq-infected Het animals treated with either CNO or saline, and mPFC-DREADD-Gq-infected Wt animals treated with saline; all groups received saline or CNO,1 h before training. Animals were trained with exposure to pairings of CS and US across four trials (CS = 5 kHz, 80 dB tone; US = 0.5-mA shock coterminating with the last second of the CS). No significant differences in learning were observed between groups across the four conditioning trials (CS4-CS1% freezing; Wt Gq saline, 44.25 ± 4.66; Het Gq saline, 38.51 ± 5.40; Het naïve saline, 38.56 ± 6.18; Het naïve CNO, 34.05 ± 4.37; Het Gq CNO, 33.25 ± 3.80; ANOVA with LSD *post hoc* test; Fig. 8). To measure fear memory retrieval, animals were subsequently exposed to the CS alone at 4 h after training (testing STM) and 24 h after training (testing LTM; Schafe and LeDoux, 2000). No significant differences were observed among the groups 4 h after training [freezing (%CS4); Wt Gq saline, 102.62 ± 8.00; Het Gq saline, 78.73 ± 5.06; Het naïve saline, 93.06 ± 15.80; Het naïve CNO, 82.96 ± 8.02; Het Gq CNO, 78.14 ± 8.27; ANOVA with LSD *post hoc* test; Fig. 8]. However, 24 h after training, mPFC-DREADD-Gq-infected Het mice treated with saline exhibited a significant deficit in their freezing response, as did noninfected Het controls treated with either saline or CNO, compared to mPFC-DREADD-Gq-infected Wt mice. In contrast, the freezing response in mPFC-DREADD-Gq-infected Het mice treated with CNO was indistinguishable from mPFC-DREADD-Gq-infected Wt mice treated with saline [freezing (%CS4); Wt Gq saline, 125.53 ± 11.42; Het Gq saline, 70.67 ± 8.42; Het naïve saline, 81.98 ± 12.23; Het naïve CNO, 87.05 ± 6.19; Het Gq CNO, 123.16 ± 16.87; ANOVA with LSD *post hoc* test; Fig. 8]. These data demonstrate that activation of the mPFC in *Mecp2* mutants can restore long-term retrieval of auditory conditioned fear memory to Wt levels, 24 h after training. Given that the duration of CNO action is ∼6 h (Whissell et al., 2016), these data indicate that transient activation of the mPFC in *Mecp2* mutants leads to a durable change in mechanisms underlying fear memory retrieval.

## Discussion

The present findings demonstrate the importance of mPFC hypofunction in RTT by showing that activation of mPFC pyramidal neurons can reverse abnormalities in breathing and long-term retrieval of conditioned fear learning in *Mecp2* Het mice, a model of RTT that recapitulates the genetic mosaicism and many phenotypic characteristics of the human disorder. It seems likely that distinct mechanisms downstream of enhanced pyramidal neuron activity underlie the reversal of these respiratory and cognitive abnormalities, respectively. This is underscored by the fact that the normalization of respiratory apneas and respiratory variability was transient and undetectable 24 h after CNO treatment, the same time point at which fear memory retrieval was rescued in mPFC-DREADD mutants. These differences could be explained by the fact that different populations of mPFC neurons innervate brainstem versus cortical and subcortical forebrain targets (Gabbott et al., 2005). In general, loss of *Mecp2* is thought to disrupt resting breathing by shifting synaptic excitatory-inhibitory balance toward increased excitability at multiple loci throughout the brainstem respiratory network (Stettner et al., 2007; Katz et al., 2009; Ramirez et al., 2013). We hypothesize, therefore, that activation of the mPFC normalizes resting breathing in Het mice by acutely modulating hyperexcitability of the brainstem network. Moreover, our data suggest that loss of cortical modulation resulting from hypoactivity of pyramidal neurons in the mPFC may underlie or contribute to behavioral dysregulation of breathing in RTT (Sceniak et al., 2015). Based on the projections of mPFC-DREADD-Gq-infected neurons, the modulation of respiratory output that we observed could have been mediated by direct corticobulbar inputs to ponto-medullary respiratory cell groups, including the nTS, VLM, and LC, and/or by projections to structures that indirectly modulate breathing, such as the hypothalamus, amygdala, and PAG. The fact that mPFC activation can normalize mutant levels of Fos expression in respiratory subnuclei of nTS suggests that cortical inputs may influence the breathing pattern in RTT, at least in part by modulating hyperexcitability of brainstem neurons involved in reflex regulation of respiratory motor output. Such a mechanism would be consistent with previous studies demonstrating that the mPFC regulates gain and sensitivity of cardiovascular reflexes mediated by the nTS (Hassan et al., 2013; Fassini et al., 2016; Scopinho et al., 2009). However, given the complexity of the brainstem respiratory network, we cannot rule out the possibility that stabilization of the breathing pattern in DREADD-activated mutants involves alterations in excitatory/inhibitory balance in structures other than the nTS as well (Abdala et al., 2016).

Further studies will be required to determine which subregion(s) of the mPFC are responsible for the rescue of Het breathing phenotypes described here, as the PL, IL, and dPC all have direct and/or indirect connections with brainstem nuclei involved in cardiorespiratory control (Gabbott et al., 2005), as well as the impact of DREADD activation of mPFC pyramidal neurons on the strength of these connections. More generally, there is growing appreciation for the fact that hypofrontality (Pratt et al., 2008), and abnormalities in the corticofugal projection network in particular contribute to the pathophysiology of diverse neuropsychiatric diseases (Shepherd, 2013). However, despite the long-standing recognition that respiratory dysfunction in RTT exhibits features of limbic cortical dysfunction (Woodyatt and Murdoch, 1996), a role for prefrontal corticofugal influences on breathing abnormalities in RTT has not previously been described. By highlighting the ability of corticofugal pathways to modulate respiratory output in *Mecp2* mutants, these findings raise the possibility that cortical influences on breathing could be harnessed to improve respiratory control in RTT.

Ongoing activity of mPFC pyramidal neurons is also critical for diverse cognitive tasks, including consolidation and retrieval of cue-dependent fear conditioning, which requires activity of neurons projecting from the mPFC to the BLA and the dMT for short- and long-term retrieval, respectively (Corcoran and Quirk, 2007; Adhikari et al., 2015; Do-Monte et al., 2015). In fact, the LTM deficit that we and others have observed in *Mecp2* mutants, 24 h after training (Stearns et al., 2007; Tai et al., 2016), is reminiscent of similar deficits described in normal mice following pharmacologic blockade of activity in the PL after training and before testing for fear memory retrieval (Corcoran and Quirk, 2007). Interestingly, our data indicate that the PL to BLA projection circuitry involved in short-term retrieval of conditioned fear memory is functional in *Mecp2* mutants whereas the PL to dMT projection circuitry required for retrieval at 24 h is impaired. Given that mPFC activation during fear conditioning restores long-term retrieval to Wt levels, our data suggest that increasing the activity or excitability of mPFC pyramidal neurons during training enables the shift in dependence from PL-BLA to PL-dMT projections that occurs by 24 h after training (Do-Monte et al., 2015).

The fact that both respiratory apneas and expression of cue-dependent conditioned fear memory can be restored to Wt levels in *Mecp2* mutants indicates that the circuitry required for these behaviors is sufficiently intact to respond appropriately to activation of pyramidal neurons in the mPFC. This finding is consistent with the fact that loss of MeCP2 results in reversible changes in neural circuit function rather than neuronal cell loss or degeneration (Armstrong et al., 1995; Bauman et al., 1995; Guy et al., 2007; Robinson et al., 2012; Lang et al., 2013; Hao et al., 2015; Lu et al., 2016). Given that loss of *Mecp2* results in functional hypoconnectivity in multiple cortical regions in addition to the mPFC (Katz et al., 2016), we predict that the approach described here is likely to be effective at ameliorating other symptoms in RTT mice as well. Moreover, these data raise the possibility that treatment strategies aimed at increasing activity in specific cortical networks may provide therapeutic benefit to RTT patients (see also Hao et al., 2015).

## Synthesis

Reviewing Editor: Esther Stoeckli, University of Zurich Decisions are customarily a result of the Reviewing Editor and the peer reviewers coming together and discussing their recommendations until a consensus is reached. When revisions are invited, a fact-based synthesis statement explaining their decision and outlining what is needed to prepare a revision will be listed below. The following reviewer(s) agreed to reveal their identity: Mathias Dutschmann.

The results reported in this manuscript demonstrate a role of the medial prefrontal cortex (mPFC) in mediating symptoms associated with Rett syndrome. In particular, excitatory hypoconnectivity in this area is prominent in Mecp2 mouse mutants which recapitulate many physiological and behavioral abnormalities observed in Rett syndrome patients. It was shown that restoring activation of pyramidal neurons within the mPFC through chemogenetic activation rescues the respiratory deficits and some of the cognitive impairments observed in Mecp2 mutants. We all agree that overall these are very interesting findings. However, several issues need to be addressed before the manuscript can be accepted:

Major points

1) DREADDGq expression mapping. Raw mages showing the spread of the infection in the mPFC and motor cortex should be included in the figures.

2) It is unclear how the authors controlled for the variability of infection rate after AAV injections. Quantification of the number of cells targeted in each injection should be included as well as images showing infected somata in the mPFC.

3) Electrophysiology. The n for the analysis of in vivo activity should be increased. An n of 2 is not rigorous enough to support the authors conclusions. It is unclear how the number of recorded cells was calculated from the extracellular recordings of spontaneous activity.

4) In Figure 4, it is unclear why only 7 animals were tested for reversibility (Het Gq Saline#2) from the 14 analyzed after CNO (Het Gq CNO).

5) The authors show that cFos activation in then nTS is elevated in Het Mecp2 mice and Gq injection normalizes the number of cFos cells. It would be informative to assess cFos expression in the mPFC and motor cortex in control conditions and after viral injection.

6) The authors suggest that different mechanisms underlie the mPFC control of respiration and cognitive abnormalities in Mecp2 het mice. Could these differences be explained by anatomically or molecularly different sets of mPFC neurons contributing to these functions?

7) Although the authors provide evidence that the mPFC projects to nTS, VLM and LC, the presence and strength of synaptic connections should be validated with synaptic tracers and electrophysiology. This limitation of the analysis should be included in the discussion.

Minor points

1) In the Methods and Material section, the authors indicate that some animals were Thy1EGFP carriers. It is unclear why some animals carried the transgene. Were the animals used in behavioral experiments backcrossed?

2) It is unclear whether the data have been tested for normality as parametric tests were used in several experiments.

3) It is unclear how sample size was determined.

4) A recent publication by Abdala et al., 2016 has shown that breathing dysfunction are strongly linked to GABAergic innervation of the pontine Kolliker-Fuse nucleus (not targeted by descending projections arising from the prefrontal cortex). Moreover, pharmacological dysinhibition of the Kolliker-Fuse nucleus disrupts the breathing pattern and swallowing activity (Rett Syndrome phenotpye!). Thus, the network mechanisms that may stabilize breathing in your study are not straight forward and this issue could be discussed in more detail.
